# Fluctuating DNA methylation sites encode colorectal tumour growth history

**DOI:** 10.64898/2026.06.04.730217

**Published:** 2026-06-09

**Authors:** Veselin Manojlovic, Calum Gabbutt, Darryl Shibata, Robert Noble

## Abstract

Determining the nature of human tumour growth is challenging given the impracticality of obtaining detailed data across time. A promising solution is to examine DNA regions whose methylation states fluctuate on clinically relevant timescales, permitting their use as high-resolution lineage tracers. However, existing methods developed for analysing the fluctuating methylation loci of normal tissue and lymphoid cancers are inapplicable to large solid tumours. Here we introduce a mechanistic computational model that tracks the evolution of heritable methylation marks as a tumour grows from a single gland to a mass of many cubic centimetres, and a coupled ABC-SMC inference workflow to estimate tumour growth parameters from multi-region bulk methylation arrays. We applied this framework to data from multiple regions of 10 resected colorectal tumours, including 3 adenomas and 7 carcinomas of diverse sizes and clinical stages. By exploring alternative models, we show that intratumour diversity, in terms of methylation errors, stems more from tumour growth via gland fission than from cell turnover within glands. Moreover, the extent of intratumour diversity varies widely between patients, mainly because of eight-fold variation in gland fission rates but also due to differences in methylation and demethylation rates. Intergland divergence patterns are consistent with neutral evolution of colorectal tumours and a cancer stem cell fraction of approximately 1%. As well as helping to resolve the nature of colorectal cancer growth and evolution, our results provide proof of principle for a method that may be adapted to other types of solid tumour.

## Full Text Availability

The license terms selected by the author(s) for this preprint version do not permit archiving in PMC. The full text is available from the preprint server.

