# Supplementary material for: Fluctuating DNA methylation sites encode colorectal tumour growth history
Source: bioRxiv. 2026 Jul 2:2026.06.04.730217. Originally published 2026 Jun 9. Preprint. [Version 2] doi: 10.64898/2026.06.04.730217 (PMC13278119; doi:10.64898/2026.06.04.730217)
Supplement: 1 [file NIHPP2026.06.04.730217V2-supplement-1.pdf]

bioRxiv preprint doi: https://doi.org/10.64898/2026.06.04.730217; this version posted July 2, 2026. The copyright holder for this preprint (which was not certified by peer review) is the author/funder, who has granted bioRxiv a license to display the preprint in perpetuity. It is made available under a CC-BY 4.0 International license.

Supporting information

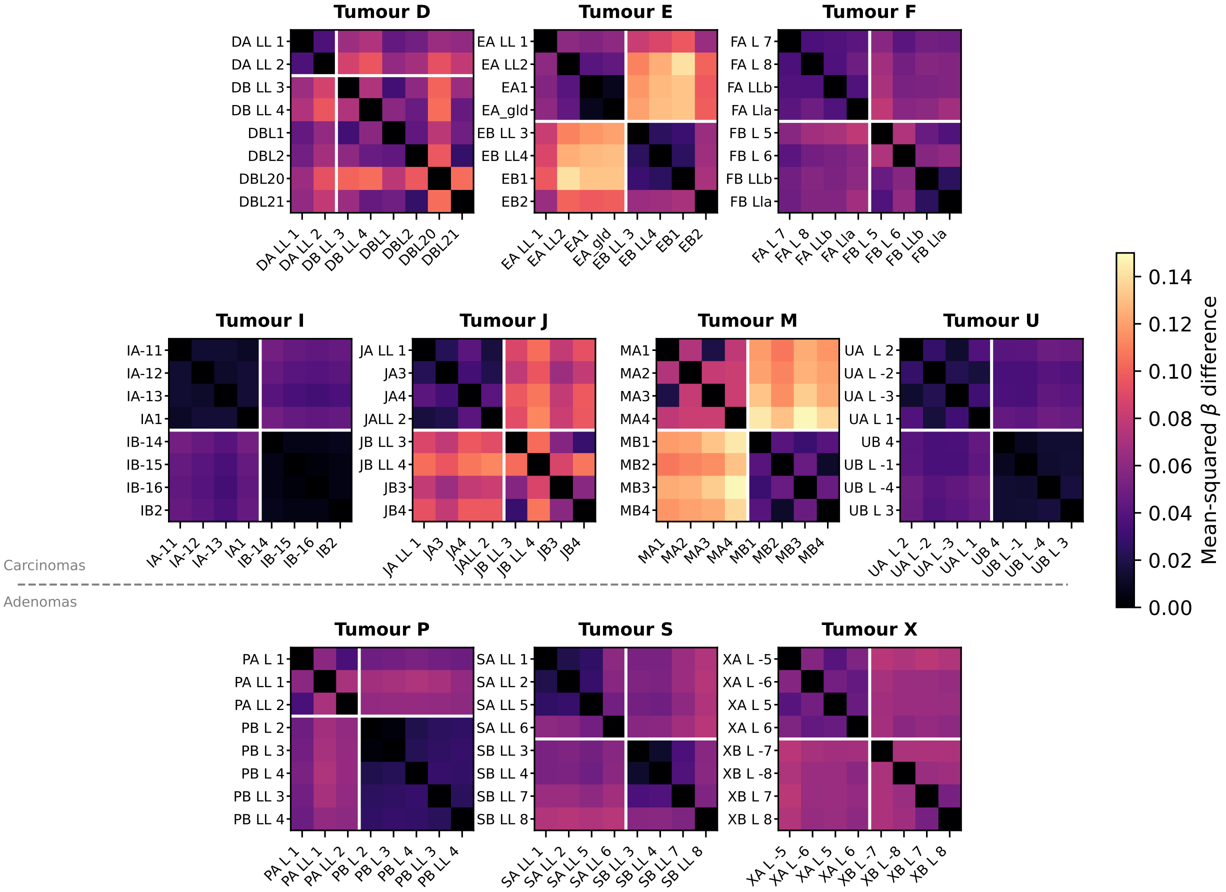

Fig S1. Inter-gland fCpG distance matrices (mean squared difference) for all 10 tumours. Bold lines separate glands from side A (top-left block) and side B (bottom-right block). Block structure is visible in all tumours, with strongest separation in tumours I, U, and M.

bioRxiv preprint doi: https://doi.org/10.64898/2026.06.04.730217; this version posted July 2, 2026. The copyright holder for this preprint (which was not certified by peer review) is the author/funder, who has granted bioRxiv a license to display the preprint in perpetuity. It is made available under a CC-BY 4.0 International license.

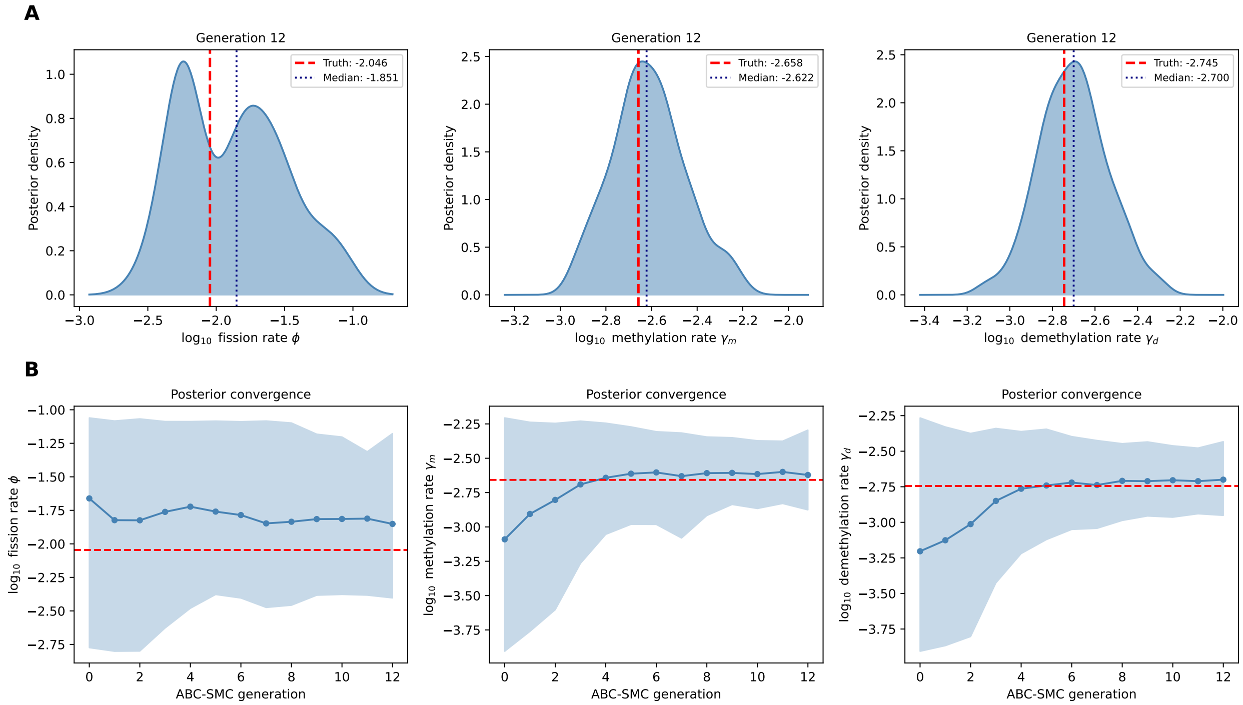

Fig S2. Synthetic parameter recovery. A: Final-generation posterior distributions (blue) for the three identifiable parameters, with ground truth values (red dashed lines). All three parameters are recovered accurately. **B:** Convergence of posterior medians and 90% credible intervals across ABC-SMC generations.

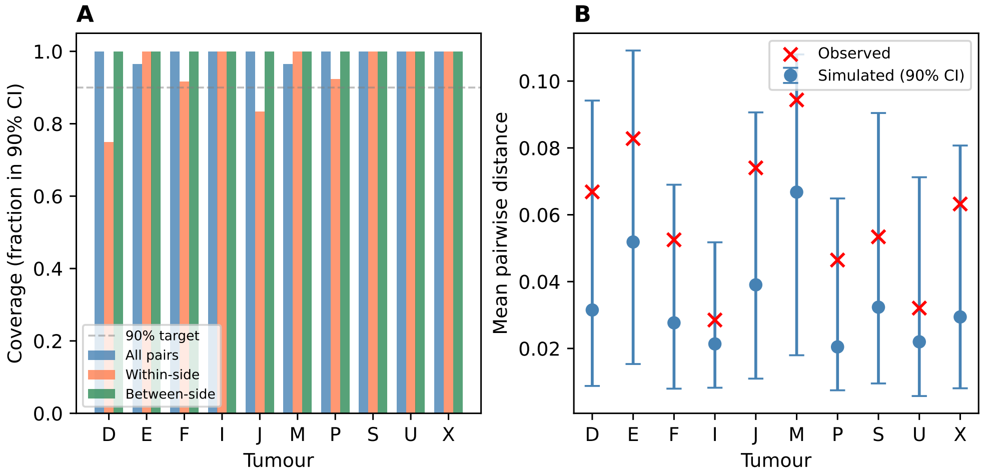

Fig S3. Posterior predictive check summary across the cohort. For each tumour, 100 datasets were simulated from the inferred posterior. **A:** Pairwise-coverage plot of the posterior-drawn simulations. **B:** Observed mean inter-gland distances. Observed values fell within the central 90% of the posterior predictive distribution for all 10 tumours.

bioRxiv preprint doi: https://doi.org/10.64898/2026.06.04.730217; this version posted July 2, 2026. The copyright holder for this preprint (which was not certified by peer review) is the author/funder, who has granted bioRxiv a license to display the preprint in perpetuity. It is made available under a CC-BY 4.0 International license.

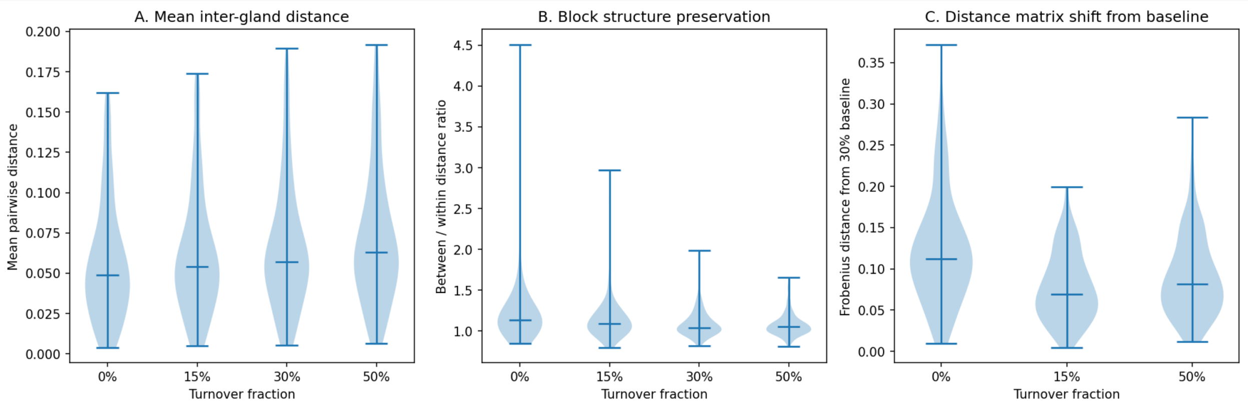

Fig S4. Model sensitivity of *methdemon* to the time spent in turnover after the growth phase as a fraction of the time spent in the growth phase. A: mean pairwise inter-gland distance across different levels of post-growth turnover. **B:** The ratio of the inter-gland distances for near and distant glands across different levels of post-growth turnover. **C:** The shift of the distance matrix across levels of post-growth turnover as compared to a 30% turnover fraction.

bioRxiv preprint doi: https://doi.org/10.64898/2026.06.04.730217; this version posted July 2, 2026. The copyright holder for this preprint (which was not certified by peer review) is the author/funder, who has granted bioRxiv a license to display the preprint in perpetuity. It is made available under a CC-BY 4.0 International license.

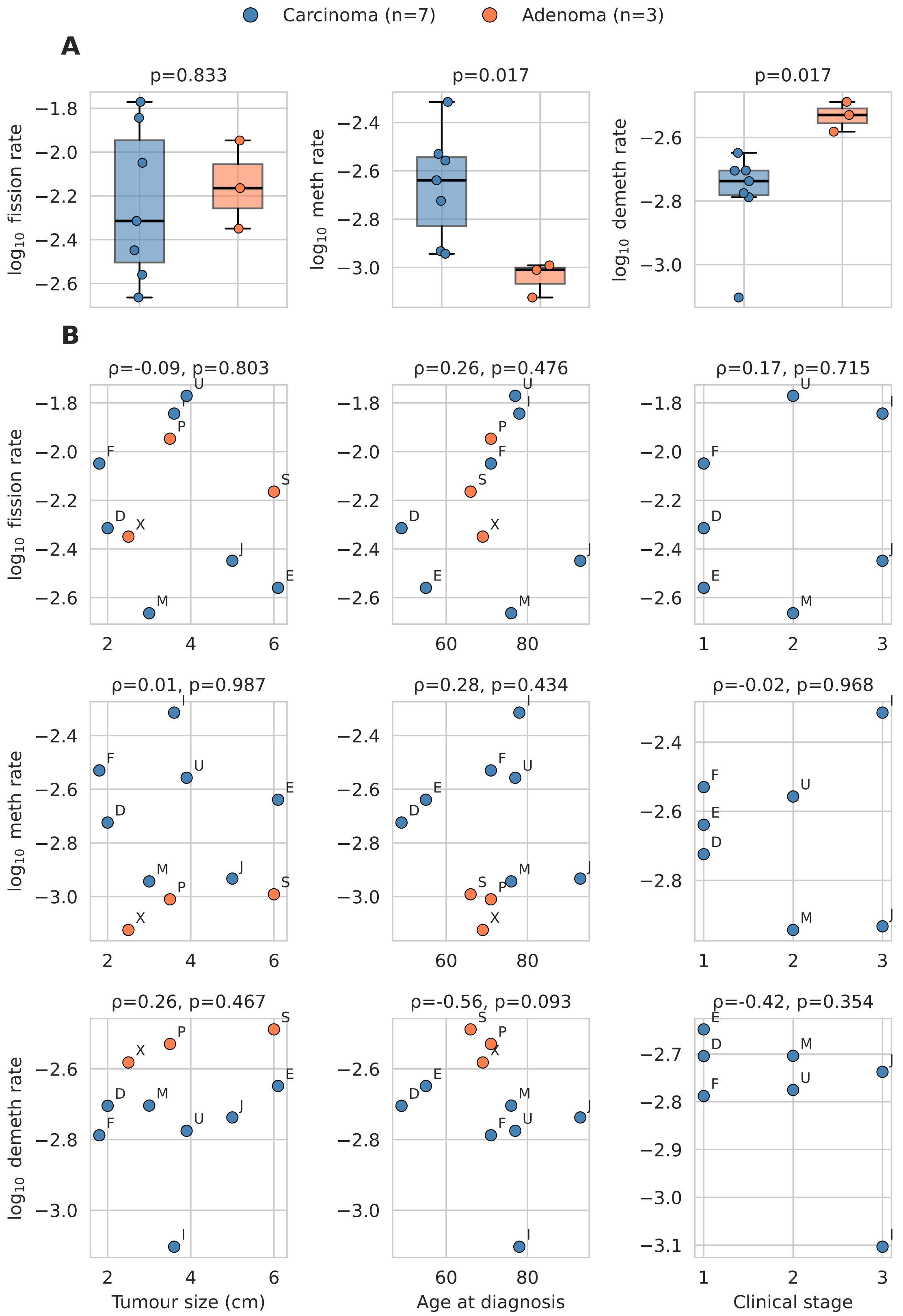

Fig S5. Analysis of how inferred parameter values vary between tumours. A: Fission, methylation, and demethylation rate comparison between carcinomas and adenomas in the cohort. **B:** Spearman correlations between inferred parameter values and clinical features.

bioRxiv preprint doi: https://doi.org/10.64898/2026.06.04.730217; this version posted July 2, 2026. The copyright holder for this preprint (which was not certified by peer review) is the author/funder, who has granted bioRxiv a license to display the preprint in perpetuity. It is made available under a CC-BY 4.0 International license.

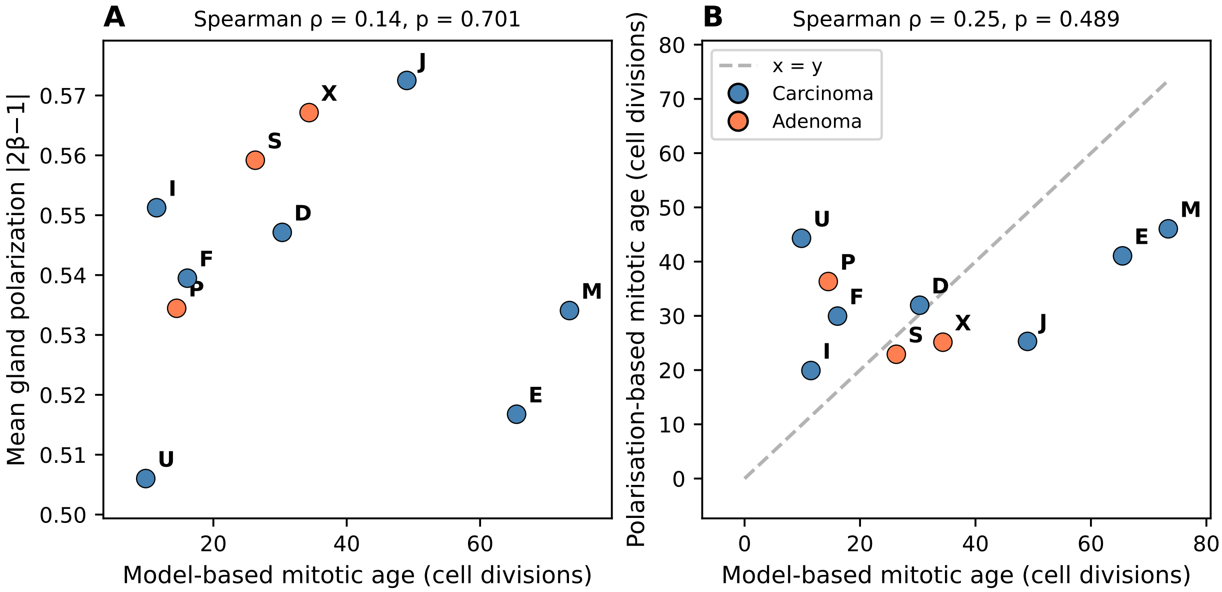

Fig S6. Consistency of mitotic age estimates. A: Mean gland polarisation versus model-based mitotic age. **B:** Polarisation-based versus model-based mitotic age estimates.
